# A Case Report of Juvenile Myasthenia Gravis; Misdiagnosis and Considerations

**DOI:** 10.1002/ccr3.73009

**Published:** 2026-06-28

**Authors:** Elaheh Heidari, Amin Saeidinia

**Affiliations:** ^1^ Faculty of Medicine Mashhad University of Medical Sciences Mashhad Iran; ^2^ Booali Research Institute Pharmacological Research Center, Mashhad University of Medical Sciences Mashhad Iran

**Keywords:** autoimmune disease, Guillain‐Barré syndrome, Juvenile Myasthenia Gravis, misdiagnosis, neuromuscular junction

## Abstract

Juvenile myasthenia gravis (JMG) is a rare autoimmune disease acquired in childhood, comprising 8%–15% of all myasthenia gravis cases depending on geographic and ethnic populations. Ocular myasthenia gravis presents as ptosis with extraocular movement restriction and is frequently misdiagnosed as third nerve palsy or congenital ptosis when bilateral. Early diagnosis prevents disease progression and unnecessary interventions. We report a case of JMG initially misdiagnosed as Guillain‐Barré syndrome (GBS) to highlight diagnostic pitfalls. A 4‐year‐old girl presented with left eye ptosis, followed by bilateral ptosis after 2 days, then gait disorder and inability to stand after 5 days. She had a previous admission 2 months earlier with severe respiratory distress, for which she received intravenous immunoglobulin (IVIG) under the suspicion of GBS. In the current admission, bilateral ptosis was evident, and repetitive nerve stimulation (RNS) showed a decremental response of > 10%, consistent with myasthenia gravis. Anti‐acetylcholine receptor (AChR) antibody was positive. She was treated with pyridostigmine (5 mg/kg/day) and showed good response. JMG should be considered in the differential diagnosis of acute muscle weakness with cranial nerve involvement, even when respiratory failure dominates, and normal cerebrospinal fluid protein in suspected GBS should prompt re‐evaluation.

## Key Clinical Message

In children with acute muscle weakness and cranial nerve involvement, consider juvenile myasthenia gravis alongside Guillain‐Barré syndrome. Targeted electrodiagnostic and antibody testing prevents misdiagnosis, enabling early treatment and better outcomes.

**Abbreviations**
AChR: acetylcholine receptor
CSF: cerebrospinal fluid
EMG: electromyography
GBS: Guillain‐Barré Syndrome
IgG: immunoglobulin G
IVIG: intravenous immunoglobulin
JMG: Juvenile myasthenia gravis
MRI: magnetic resonance imaging
NCV: nerve conduction velocity
PICU: pediatric intensive care unit
RNS: Repetitive nerve stimulation

## 1 Introduction

Juvenile Myasthenia Gravis (JMG) is a rare autoimmune disorder affecting the postsynaptic neuromuscular junction. The estimated annual incidence is 1–5 per million children, though higher rates (up to 10 per million) have been reported in some Asian and Middle Eastern populations [1, 2]. JMG is characterized by fatigable muscle weakness, typically presenting with ocular symptoms such as ptosis and diplopia, which generalizes in over half of pediatric cases [3]. The pathogenesis predominantly involves immunoglobulin G (IgG) antibodies targeting the acetylcholine receptor (AChR), leading to complement‐mediated damage and impaired synaptic transmission [4].

The diagnostic journey for JMG is often protracted, with a well‐documented risk of misdiagnosis due to its phenotypic heterogeneity and fluctuating symptom course [5]. Young children, in particular, pose a diagnostic challenge as they may lack the ability to articulate symptoms like diplopia or generalized fatigue [3]. In acute presentations with rapid progression, JMG is frequently mistaken for other neurological emergencies, most notably Guillain‐Barré Syndrome (GBS). Several case reports have documented similar misdiagnoses of JMG as GBS, particularly when respiratory failure precedes oculobulbar symptoms [5, 6, 7]. This misdiagnosis carries significant iatrogenic risk, as first‐line treatments for GBS, such as plasmapheresis, are also used for myasthenic crisis, potentially masking the true diagnosis and delaying definitive long‐term immunosuppressive therapy [7]. The rationale for this report is to highlight this specific diagnostic pitfall and provide clinicians with a practical framework to distinguish JMG from GBS in acutely ill children.

## 2 Case History/Examination

A 4‐year‐old previously healthy girl was presented by sudden ptosis in the left eye and then it developed in both eyes after 2 days. It had then gait disorder and inability to stand after 5 days. The family has no history of consumption of contaminated home‐canned foods and.vegetables or traditional seafood. All other members were healthy. She had no fever, nausea and vomiting, headache, history of trauma, preceding upper respiratory tract infection, respiratory distress and swallowing problems, and dysphagia. Level of consciousness was normal with no evidence of facial muscles' weakness, diplopia, history of sialorrhea or periodic inability to hold neck up, altered speaking and chewing problem. No wasting or fasciculation of any muscle was seen. She had a history of previous admission 2 months ago because of the same. Presentation with severe respiratory distress requiring mechanical ventilation.

## 3 Differential Diagnosis, Investigations, and Treatment

She was received intravenous immunoglobulin (IVIG) with suspicious to Guillen Barret syndrome (GBS) in that admission. She underwent lumbar puncture with results of normal pattern and normal magnetic resonance imaging (MRI) was reported. According to severe respiratory distress and sialorrhea, she was admitted to the pediatric intensive care unit (PICU) and underwent mechanical ventilation. After weaning patients and in the diagnostic process, she had normal electromyography (EMG) and nerve conduction velocity (NCV) that did not confirm the GBS.

## 4 Conclusion and Results

**FIGURE 1:**
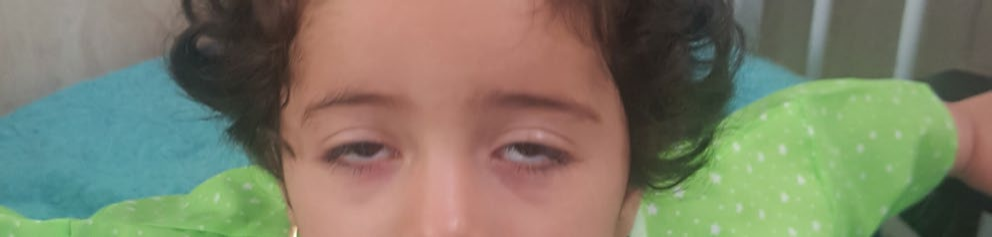
Bilateral ptosis in a 4‐year‐old girl during initial presentation.

**FIGURE 2:**
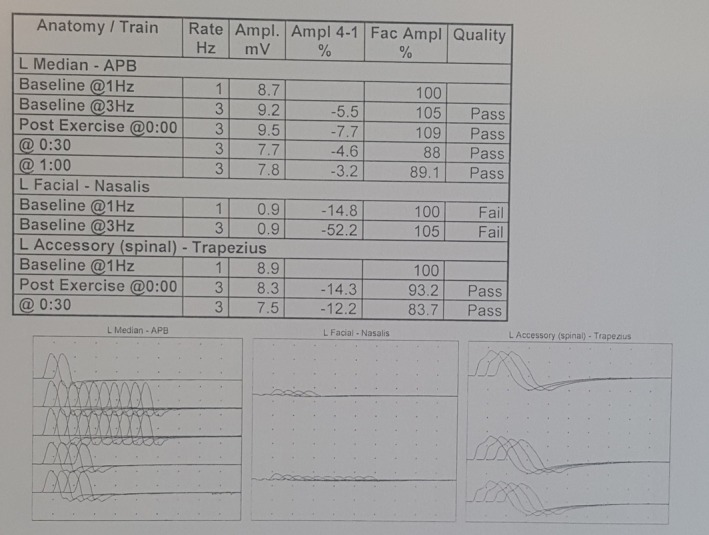
Repetitive nerve stimulation (RNS) studies showing a decremental response > 10% at low‐frequency (3 Hz) stimulation, consistent with a postsynaptic neuromuscular junction disorder.

In current admission, the initial vital sign was blood pressure 80/60 mmHg, heart rate: 90/min, respiratory rate: 32/min, oxygen saturation: 98% and axillary temperature: 37.1°C. There were no significant findings in skin, heart, lung, and abdominal examinations. Ptosis in both eyes were obvious (Figure 1) with no decrease in visual field, eye movement limitation, and diplopia. Fundus oculi were normal, and speech was not affected. She had no problem in gag reflex. In limbs examination, there was no decrease in range of motion, deep tendon reflex, edema, and abnormal plantar reflex. There was abnormal gait with non‐weight bearing. She was admitted for more workup. Lumbar and brain MRI was performed and there was no pathologic finding. She underwent lumbar puncture and cerebrospinal fluid was normal (WBC = 0, RBC = 0, sugar = 104, protein = 10, LDH = 115). Urine analysis for toxicology was normal. EMG‐NCV in this admission demonstrated a significant decrementing pattern (> 10% decrement at 3 Hz stimulation) on low frequency RNS study of facial and spinal accessory nerves that was consistent with myasthenia gravis (Figure 2). There were no other electrolyte or laboratory abnormalities. Anti‐AchE receptor antibody was requested and was positive. She was treated with Mestinon (Pyridostigmine) (5 mg/kg/day) and showed good response.

## 5 Discussion

This report elucidates a severe case of anti‐AChR positive JMG in a preschool‐aged child, whose initial presentation with acute respiratory failure was misdiagnosed as GBS. The case underscores a critical diagnostic pitfall in pediatric neurology and highlights several key learning points to enhance early recognition and accurate management of JMG.

The initial misdiagnosis of GBS, while understandable in the context of acute respiratory distress requiring mechanical ventilation, was inconsistent with several paraclinical findings. A hallmark feature of GBS is albuminocytological dissociation in the cerebrospinal fluid (CSF), characterized by elevated protein levels with a normal white cell count, which is found in over 80% of patients after the first week of symptoms [8]. Our patient's completely normal CSF protein level (10 mg/dL) during the first admission was a significant red flag that should have prompted immediate re‐evaluation of the GBS diagnosis. Furthermore, the absence of electrophysiological evidence for a demyelinating or axonal neuropathy on NCV/EMG, the cornerstone for confirming GBS [9], provided compelling evidence against the initial diagnosis. The positive response to IVIG, while effective in GBS, is non‐specific and also a standard therapy for myasthenic crisis, thus providing a misleading confirmatory bias [10].

The recurrent, fluctuating nature of the symptoms is the quintessential hallmark of an autoimmune disorder like JMG rather than a monophasic illness like GBS. The second admission, with its clear progression from ptosis to generalized weakness, provided the classic phenotypic clue. The definitive diagnosis was established through targeted neurophysiology. Repetitive nerve stimulation (RNS) is a highly specific test for postsynaptic neuromuscular junction disorders, with a decremental response of > 10% being diagnostic [4]. Performing RNS on facial and spinal accessory nerves, as done in this case, increases the sensitivity of the test in generalized JMG [11]. This, coupled with the positive anti‐AChR antibody assay, which has a specificity nearing 100% for autoimmune myasthenia gravis [12], confirmed the diagnosis beyond doubt.

This report has several limitations. First, as a single case report, the findings may not be generalizable to all JMG presentations. Second, long‐term follow‐up data on this patient's response to pyridostigmine and potential need for immunosuppression are not yet available. Third, genetic testing to exclude congenital myasthenic syndromes was not performed. Fourth, the facial image (Figure 1) may limit anonymity despite informed consent; readers should be aware that identifiable features are published with explicit parental permission.

This case highlights two crucial considerations for pediatricians, neurologists, and intensivists: JMG is a Master Mimic. Myasthenic crisis can present identically to other causes of acute neuromuscular respiratory failure, including GBS and botulism. A thorough history looking for prior fluctuating weakness or ocular symptoms is essential, even in young children.

Atypical Features Demand Re‐evaluation: Normal results that contradict the working diagnosis (e.g., normal CSF protein in suspected GBS, normal NCV in suspected neuropathy) must not be dismissed. They should be recognized as critical clues necessitating a diagnostic pivot.

This case underscores the imperative for a high index of suspicion for Juvenile Myasthenia Gravis in any child presenting with acute flaccid weakness or respiratory failure. Misdiagnosis, particularly as GBS, leads to delays in initiating appropriate long‐term immunotherapy and exposes the patient to the risk of recurrent crisis. A meticulous diagnostic approach must prioritize the nuanced interpretation of paraclinical data—especially normal results that contradict the initial hypothesis—and should include RNS of cranial nerves when JMG is suspected. Enhanced awareness of these diagnostic pitfalls is essential to improve outcomes for children with this treatable autoimmune disorder.

## Author Contributions

**Elaheh Heidari:** conceptualization, data curation, investigation, writing – original draft, writing – review and editing. **Amin Saeidinia:** conceptualization, data curation, writing – original draft, writing – review and editing.

## Funding

The authors have nothing to report.

## Ethics Statement

Publishing this case presentation is performed according to ethics guidelines of Mashhad University of Medical Sciences. Patient was evaluated for her problem and her parents fulfilled informed consent for participation.

## Consent

Written informed consent for the publication of this case report and any accompanying images was obtained from the patient's legal guardian (his parents). The consent form included permission to publish clinical data and imaging details in accordance with the journal's policies on patient confidentiality and privacy. A copy of the signed consent is available to the editor upon request. The guardians provided consent on behalf of the child in accordance with the journal's policies on the publication of identifiable patient information. For Figure **1**, which shows identifiable facial features, specific additional consent was obtained.

## Conflicts of Interest

The authors declare no conflicts of interest.

## Data Availability

The data that support the findings of this study are available from Ghaem Hospital, Mashhad University of Medical Sciences, but restrictions apply to the availability of these data, which were used under license for the current study, and so are not publicly available. Data are however available from the authors upon reasonable request and with permission of the corresponding author.
